# Who sends the message matters: social media messengers and adolescent eating

**DOI:** 10.3389/fnut.2026.1799978

**Published:** 2026-05-04

**Authors:** Yara Qutteina, Lotte Hallez, Paulien Decorte, Charlotte De Backer, Tim Smits

**Affiliations:** 1 Media, Information and Persuasion Lab, KU Leuven, Leuven, Belgium; 2 Department of Communication Sciences, University of Antwerp, Antwerp, Belgium

**Keywords:** adolescent, eating, food marketing, message exposure, message source, social media

## Abstract

Adolescents encounter large volumes of non-core (i.e., nutritionally poor and unhealthy) food messages on social media. The relationship between these messages and eating is a product of both the volume of exposure to the message (e.g., frequent exposure) and the various sources (i.e., messengers) relaying each of these messages. This study investigates and compares how adolescents’ perceived volume of exposure to food messages from different social media messenger categories (peers, influencers, celebrities, food brands, and health organizations) is associated with their eating outcomes. A cross-sectional survey of 1,002 adolescents (aged 11–19 years) showed that adolescents report significantly higher exposure to non-core food messages posted by peers, celebrities, influencers, and brands compared to core food messages, and that greater exposure to such messages is significantly associated with higher non-core food liking, norms and/or intake. Only exposure to core food messages posted by health organizations was significantly associated with core food consumption. These findings highlight the importance of food marketing regulations particularly those addressing peer and influencer driven content. They also highlight the significance of peers and health organizations, as messenger categories, in social media food communication to adolescents.

## 1 Introduction

The persuasiveness of food communication, including food marketing, is a product of the joint effect of exposure (i.e., how much and how frequently one is exposed to a message, also referred to as volume of exposure) and power (i.e., the persuasive characteristics embedded in the content of each message) (1). Much research investigated and found a positive relationship between volume of exposure to a food message and eating. For example, Gascoyne et al. (2) found that adolescents who report at least once-a-week exposure to food advertisements on social media were significantly more likely to also report increased consumption of non-core food (i.e., high-energy low-nutrient food). Similarly, much research also investigated the different characteristics of a single food message which persuade one to like or eat the food depicted in that message. For example, Folkvord et al. (3) demonstrated that characteristics of the message source, such as the use of a real versus fictitious social media influencer and the degree of parasocial interaction, significantly shape food-related attitudes and behavioral intentions, highlighting the persuasive role of message-level features beyond exposure volume. However, there is a lack of research that considers the joint exposure and power of the (social media) food message in relation to eating. As such, this study will focus on both the volume of exposure and the source of the message (as a persuasiveness characteristic and power of the individual message) in investigating the relationship between social media food message exposure and adolescent eating.

In today’s media life, exposure to social media food messages can vary tremendously between individuals. Previous evidence showed that repeated exposure to food messages will have a favorable effect on the attitude or consumption of the food communicated therein (4). Such effects are attributed to *mere-exposure* (5), where repeated exposure to certain foods lead an adolescent to develop favorable attitudes towards these foods. The mere exposure phenomenon is further elaborated on in the *perceptual fluency model*, which predicts that repeated exposure to food messages will make these messages more accessible in an adolescent’s memory (6). More accessible messages in one’s memory will then facilitate the ease and speed at which these messages are processed creating increased familiarity and positive attitudes towards the communicated food, eventually leading to increased food choices and consumption (6). In this context, repeated exposure to food messages is considered as a peripheral cue that is processed by an adolescent’s *System 1* thinking (4). This peripheral System 1 processing is characterized by implicit learning that is unconscious, innate and automatic (7). When an adolescent repeatedly encounters a food message, the repetitive format (rather than the content) of the message is processed (4). When this food peripheral cue (i.e., repetition) is combined with the nature of covert persuasive messages on social media and adolescents’ developmental immaturity, adolescents are then processing food messages using lower levels of persuasion processing paving the way for food attitude and intake changes (8).

In addition to the exposure of the message, this research will also consider the power of the content of the message itself. According to McGuire’s (9, 10) *Communication/Persuasion Model* the persuasiveness of a message is the product of a number of input communication factors, such as the source of the message (or messenger, e.g., peers & influencers). Different qualities of different messengers affect how well the message is being received and determine its persuasive outcome. Such messenger qualities include demographic characteristics, credibility, attractiveness, similarity with the adolescent receiver, among others.

The social media food messages that reach adolescents are mostly posted by peers, followed by brands, social media influencers, and celebrities (11). These messages mostly display non-core food, including marketed and non-marketed content, and thus explicitly or implicitly promote and normalize the consumption of non-core food (11, 12). It remains unclear whether the social media food messages communicated by these different messengers (peers, social media influencers, celebrities, and brands) are similarly or differently related to adolescents’ eating outcomes. Accordingly, this study will investigate the power of the food message by comparing between social media messengers in terms of their relation to adolescent eating.

### 1.1 The significance of peers in steering adolescent eating

Peers are an important message source and factor to consider when studying adolescent choices and behavior. During adolescence, parental influence decreases and is slowly replaced by peer influence (13). Peers, i.e., members of the adolescent’s social network who are similar in age (14), become important at the microsystem level in influencing adolescents’ decisions and choices, including those related to eating (15). Peers also become integral to the social environment adolescents may seek to conform to, greatly influencing adolescents’ perceived norms and behavior (16). In addition, peers are perceived by adolescents as a similar message source. According to McGuire’s Communication/Persuasion Model (9), this perceived similarity between the message source and the recipient is an additional factor that enhances peers’ persuasiveness. Yet, peers are often found to encourage adolescents to eat non-core food instead of core food (15).

### 1.2 The role of celebrities in influencing adolescent eating

Traditional celebrities, who gained fame outside of social media, have long been known to have an influence on consumers. Brands, particularly those marketing non-core food, have been benefiting from the influence of celebrity endorsements for decades, especially for the endorsement of non-core foods and beverages (17, 18). Celebrity endorsers’ persuasive power in part stems from adolescents’ parasocial interactions (i.e., illusionary social experiences) which form parasocial relationships (i.e., illusionary social relationships) with celebrities (19).

### 1.3 The role of social media influencers in shaping adolescent eating

The presence of social media influencers has grown and the extent of their influence on adolescents is becoming more obvious as research on this topic progresses. Social media influencers are community leaders who manifest as peers and brand marketers at the same time. Similar to celebrities, adolescents form parasocial relationships with these influencers (3, 19). Unlike the one-way communication between traditional celebrities and adolescents, influencers rely on a two-way communication with their adolescent followers as they regularly engage and communicate with followers on social media (19). Furthermore, adolescents may consider a social media influencer as a peer, which could exacerbate the impact of influencers on these adolescents (9, 20). As such, adolescents typically perceive social media influencers as more credible, trustworthy and authentic compared to traditional celebrities or food brands (20).

### 1.4 Adolescents’ exposure to marketing by food brands

Evidence confirms that food marketing (e.g., television and billboard ads) influences adolescent eating (21). For example, Murphy et al. (22) show that adolescents recall and recognize unhealthy brands better on social media and were more likely to share them. On social media, food brands benefit from new opportunities of personalized and authentic marketing strategies. Beyond celebrity and influencer endorsements, brands act as direct message sources by disseminating food marketing content through their own social media pages, posting personalized advertisements, launching competitions, providing links to external websites, among others.

### 1.5 Adolescents’ exposure to core food messages by health organizations

Similar to brands, health professionals and health organizations (including governmental health departments, health non-governmental organizations, etc.) have noted the popularity of social media and its value in accessing individuals (23). These health organizations have adopted social media as a platform to communicate health information. However, their use of social media is somewhat superficial as compared to food brands (23, 24). For example, Thackeray et al. (25) found that American public health departments only use social media to post information without interacting with users as brands do. This finding is mirrored in the limited research assessing health organizations’ use of social media (24). Despite their limited use of social media, health organizations are perceived as a credible source of information (26). According to the Persuasion/Communication model, such a perception can increase the persuasiveness of the messages they expose adolescents to on social media (9).

Beyond message and source characteristics, persuasion outcomes are also shaped by audience factors, which are particularly relevant in the case of adolescents. Adolescence is a developmental period marked by rapid cognitive, emotional, and social change, including increases in impulsivity and reduced inhibitory control, which heighten sensitivity to persuasive food cues in social media environments (27). At the same time, adolescents experience growing autonomy in food choices and reduced monitoring by parents, making them more susceptible to external influences such as marketing and peer norms (13). Adding to their vulnerability, current (self-)regulatory approaches—such as the EU Pledge and the Belgian Food Advertising Code (28, 29)—remain weak, rely heavily on voluntary compliance, and have historically excluded adolescents from meaningful protection, leaving them disproportionately exposed to unhealthy food marketing, particularly online. These vulnerabilities co-occur with substantial individual variability: age, sex, and personal traits (such as self-regulation, motivation) can influence how messages are processed and how persuasive attempts influence behavior (30). For instance, research grounded in Self-Determination Theory highlights that adolescents differ in their degree of self-regulation, which can shape responsiveness to informational versus controlling cues in food communication (31).

### 1.6 Research questions and hypotheses

The food messages adolescents are exposed to on social media vary in their exposure and power (e.g., message source). Considering the significance of the volume of food message exposure in adolescent persuasion as well as the different sources of core and non-core food messages on social media, the aim of this study is to investigate the associations between perceived volume (i.e., frequency) of exposure to food messages by different social media messengers (i.e., message source as one indication of message power) and eating outcomes among adolescents 11–19 years old. This age range aligns with standard international definitions of adolescence (e.g., see WHO (32) and Sawyer (33)). To achieve this, we will utilize the same large dataset used in a previous study (34). In that study, the association between overall self-reported exposure to social media food messages and eating outcomes among adolescents was investigated, with a focus on the mediation role of perceived norms and food literacy. However, what was missing in the previous study (34), was an investigation of the combination of exposure and power of these social media food messages, particularly in terms of comparing different volumes of perceived exposure to various messenger categories and how these relate to adolescent eating outcomes. Therefore, the current study will address this gap and focus on the sources (messenger categories) of these social media messages while taking into account the volume of exposure adolescents perceive and report. As such, we first assess adolescents’ perceived frequency of exposure to food messages posted by different social media messengers: (RQ1) Are there significant differences in adolescents’ perceived volume of exposure to core and non-core food messages across different messenger categories (namely peers, celebrities, influencers, and brands) on social media? Given that previous literature demonstrates that generally adolescents are significantly exposed to higher non-core food messages on social media as compared to core food messages, we hypothesize that this is the case regardless of who the source of the messages is. In other words, we hypothesize that (H1) adolescents will report significantly higher exposure to non-core food messages, as compared to core food messages, posted by peers (H1a), traditional celebrities (H1b), social media influencers (H1c), and brands (H1d).

To investigate how exposure to food messages from different social media sources is associated with eating, we assess multiple outcomes including adolescents’ eating attitudes, perceived norms, intentions to eat, and food intake. Accordingly, the second research question (RQ2) is: what are the relationships between perceived volume of exposure to food messages posted by various messenger categories on social media and eating outcomes among adolescents? We hypothesize that (H2) higher self-reported exposure to non-core food messages by peers (H2a), traditional celebrities (H2b), social media influencers (H2c), and brands (H2d) will be associated with increased non-core food attitudes, perceived norms, eating intentions, and intake among adolescents. In this study we differentiate between two types of perceived norms: beliefs about what others typically eat (also known as descriptive norms) or what others believe are healthy foods to eat (also known as injunctive norms).

Food literacy (i.e., attitudes, conceptions, skills, and behaviors needed to make decisions and choices that mediate balanced healthy diets (35)) plays an important mediating role in the relationship between core food exposure and intake (34). As such, this variable will be investigated in relation to core food exposure. Thus, we hypothesize that (H3) higher self-reported exposure to core food messages by peers (H3a), traditional celebrities (H3b), social media influencers (H3c), and brands (H3d) will be associated with increased core food attitudes, perceived norms, eating intentions and intake as well as higher food literacy among adolescents. In this study we will also explore adolescents’ exposure to core food messages posted by health organizations acting as social media messengers. Finally, we will also compare the different messengers on social media to determine (RQ3) which social media messengers, in terms of adolescents’ perceived volumes of exposure to core and non-core food messages, have the strongest association with core and non-core food intake among adolescents?

## 2 Materials and methods

### 2.1 Design and participants

To assess the relationship between social media food messages and adolescent eating, we conducted a cross-sectional survey among 1,009 adolescents 11–19 years old, between October and December 2019. Using the complete official registry of secondary schools in Flanders, we constructed the sampling frame and selected schools through multistage cluster sampling. The selected school sample was stratified by type of education (i.e., general, vocational, and technical) to ensure representation across the main educational tracks in Flanders. Given that school-level response rates are typically low (e.g., (36)), we oversampled at the school level to ensure adequate participation and randomly selected 140 schools to invite for participation. In total, 18 schools across Flanders agreed to participate and were included in the study. Each participating school provided a minimum of two classes and the classes were distributed across school levels to capture the full age range of adolescents. To test the survey protocol and the questionnaire content, a pilot with 300 Flemish adolescents was conducted. Following the pilot and the attainment of written informed (parental) consent and assent, adolescent participants were recruited. In each selected class, during a scheduled class period and onsite, two trained researchers, in the presence of a teacher, introduced the study to students as a group and provided each student with a digital consent/assent link. Students who consented completed the Qualtrics-programmed questionnaire individually during the same session, while those who declined to participate or whose parents had not provided parental consent prior to data collection were given a quiet alternative task. The research protocol was reviewed and approved by the first author’s university’s board for ethical review (G-2018061257).

### 2.2 Materials and tools

The majority of the scales in this study (i.e., the scales measuring food marketing exposure, intention to eat, food attitudes, and perceived norms) were measured in reference to six food categories, which were later during analysis adapted into scales specific to core or non-core food. This categorization was based on the definitions of Kellet et al. (37) and Toumpakari et al. (38) who classify core food as those belonging to the main dietary guidelines food groups including fruits, and vegetables. Food not belonging to any of these groups and that is typically high in energy yet low in nutrients such as candy, fried chicken, soft drinks, and cakes are classified as non-core food (37, 38). Following this classification, the categories included under core foods were water and unsweetened drinks, fruits and vegetables, whereas the non-core foods included sweetened drinks, sweets and (salty)savory snacks, as well as junk food. All food categories were explained and accompanied by examples to aid participants’ interpretation (see OSF for more details).

The questionnaire included 36 items measuring exposure to social media core and non-core food messages. Participants reported the extent to which they saw food messages posted by peers, influencers, celebrities, brands and health organizations, on a 7-point scale ranging from ‘not at all’ to ‘very often’. An explanation/definition was provided for each messenger category. The scores for each messenger source were calculated in reference to core and non-core food to generate three scores (i.e., total food message exposure, core food message exposure, and non-core food message exposure) that ranged from a low score of 1 to a high score of 7.

Eating outcomes included food attitudes, norms, literacy and intake. Attitudes comprised of food liking, assessed by asking participants to report their recollected liking of each food, and perceptions of food healthiness, assessed by asking participants how healthy they thought each food item was. Both outcomes were measured on 5-point Likert scales adapted from Dixon et al. (39). Perceived norms measurements were divided into descriptive (how often do you think others typically eat these foods?) and injunctive (how healthy do others think it is to eat these foods?) norms. Both perceived norms were measured using 5-point Likert adapted from Dixon et al. (39). As for food literacy, a validated 5-point Likert scale measured different areas of food literacy including daily food planning, food preparation skills, food label examination, health snack styles, and resilience (40). Additionally, a validated food frequency questionnaire measured food intake by assessing how often and in what amounts adoelscents consumed different types of foods over the past month, including core foods (e.g., water and unsweetened beverages, fruits, and vegetables) and non-core foods (e.g., sweetened drinks, sweets and savory snacks, and junk food) (41).

A number of secondary variables were also measured. Intention to eat was measured by asking participants how much food they intend to eat in the next month compared to how much they eat now, using a 5-point Likert scale ranging from “much less” to “much more” (39). This scale included the 6 categories of core and non-core food described above. Self-regulated autonomy and adolescents’ motivation to eat core food was measured using the validated treatment self-regulation questionnaire (TSR) (42). Additionally, the questionnaire collected information on gender, age, socioeconomic status (SES, assessed by mother’s highest level of education and type of education), parental support (measuring the extent to which both mother and father support healthy eating), and height and weight (used to calculate age-adjusted Body Mass Index) (43–45), see suplementary material for further details.

### 2.3 Analysis

R software 2019 (R version 3.6.1) was used to carry out data analysis (46). Prior to analysis, the dataset was cleaned by excluding invalid responses, such as illogical answers in open-ended items and evidence of straightlining. To answer RQ1, sign test analysis was used to determine the difference in median scores between self-reported exposure to core and non-core food messages on social media. To answer RQ2, Kendall’s rank correlation or Kruskal–Wallis tests were used to assess bivariate relationships between reported exposure to social media food messages and the different self-reported eating outcomes including food intake, attitudes, perceived norms and food literacy, whereby all analysis assumptions were met. To answer RQ3, two multiple multivariate regression models per messenger source (i.e., peers, traditional celebrities, social media influencers, and brands), were analyzed to assess the relationship between: 1. reported exposure to core social media food messages as an exposure variable and multiple core food intake outcome variables (i.e., water and unsweetened drinks, fruits and vegetables), 2. reported exposure to non-core social media food messages as an exposure variable and multiple non-core food intake outcome variables (i.e., sweetened drinks, sweets and savory snacks, and junk food). As for health organizations, only one multiple multivariate regression model was developed to assess the relationship between reported exposure to core social media food messages and multiple core food intake variables. The models controlled for the following covariates: self-regulated autonomy (TSR), intentions to eat, gender, and BMI-for-age. These variables were selected based on empirical evidence that shows that individual characteristics such as self-regulation, behavioral intentions, gender and weight status are correlates of eating related behavior (47–51). Including these covariates also aligns with health behavior theoretical frameworks (31) that highlight key sources of individual variation, such as self-regulation and eating intentions, that may confound associations between perceived exposure and eating. The models were also tested with the inclusion of other covariates identified as relevant in the literature, namely perceived descriptive norms, injunctive norms, food literacy, age, SES and parental support (31, 47, 50). However, these factors often did not improve the model and (mostly due to missing values for SES) greatly reduced the sample size, due to which they were not included in the reported analysis.

## 3 Results

Eleven- to nineteen-year-old Flemish adolescents (*N* = 1,098) from Belgium participated in the survey. Following cleaning, a total of 1,002 participants were included in the study. On average participants were 15 years old (*M* = 15, SD = 2.06) and the majority (58%) were females. Further descriptive characteristics of the sample can be found in Qutteina et al. (34).

The first research question inquired about the presence of differences in adolescents’ perceived exposure to core and non-core food social media messages posted by different messengers. As demonstrated in Table 1, adolescents reported significantly higher exposure to non-core food messages than core foods on social media posted by peers (*S* = 42, *p* < 0.001), traditional celebrities (*S* = 171, *p* < 0.001), social media influencers (*S* = 141, *p* < 0.001), and food brands (*S* = 79, *p* < 0.001). This confirms H1a, H1b, H1c and H1d.

**Table 1:** Mean, median (on scales of 1–7) and standard deviation of adolescents’ self-reported exposure to social media food messages, and the median differences between exposure to core and non-core food messages (based on sign tests, *N* = 1,002).

Food message source	Mean	Median	SD	Difference in exposure to core and non-core food messages
Peers				
Overall food	2.928	3.000	1.890	*S* = 42, *p* < 0.001
Core food	1.993	1.667	1.866	
Non-core food	3.780	3.667	1.1361	
Traditional celebrities				
Overall food	3.745	4.000	1.825	*S* = 171, *p* < 0.001
Core food	2.765	2.667	1.473	
Non-core food	3.667	3.749	1.609	
Social media influencers				
Overall food	3.785	4.000	1.870	*S* = 141, *p* < 0.001
Core food	2.702	2.333	1.462	
Non-core food	3.878	4.000	1.617	
Food brands				
Overall food	4.886	5.000	1.918	*S* = 79, *p* < 0.001
Core food	3.104	3.000	1.463	
Non-core food	4.751	5.000	1.564	
Health organizations				
Core food	3.581	3.333	1.697	NA

A closer examination of the descriptive values in Table 1 reveals that perceived volume of exposure to food messages seems to differ across the four social media messengers. Adolescent participants reported seeing core foods most often posted by health organizations (*M* = 3.58, SD = 1.70), followed by brands (*M* = 3.10, SD = 1.46). Core foods were least often perceived to originate from peers (*M* = 1.99, SD = 1.87). A further exploration using sign test analysis demonstrated that the differences in these descriptive values were significant. Self-reported exposure to core food messages differed significantly across the four social media messengers (*S* = 195–636, *p* < 0.001), with the exception of the comparison between social media influencers and celebrities (*S* = 351, *p* = 0.066), see Supplementary Table 1. For non-core food messages, the picture is radically different: These are most frequently attributed to brands and influencers, while exposure to non-core food messages from celebrities and peers seemed to be lower, see Table 1. Additional sign test analysis confirmed that perceived exposure to non-core food messages differed significantly across social media messengers types (*S* = 202–578, *p* < 0.001), except for the comparisons between peers and traditional celebrities (*S* = 421, *p* = 0.518), and between peers and social media influencers (*S* = 440, *p* = 0.137), see Supplementary Table 2 for more details.

### 3.1 Non-core food social media sources and how they relate to food outcomes and perceptions

The second research question examined the relationship between the perceived volume of exposure to core and non-core food messages from different social media sources and adolescents’ eating outcomes. The following section will partially answer this question by examining associations between various eating outcomes and perceived volume of exposure to non-core food messages from four sources: peers, traditional celebrities, social media influencers, and brands (see Table 2).

**Table 2:** Kendall’s rank correlation coefficients depicting associations between perceived volume of exposure to non-core food messages (from different social media messengers) and non-core food related eating outcomes among adolescents.

Social media messenger	Food liking	Perceived healthiness	Descriptive norms	Injunctive norms	Intention to eat
Peers	3.65^***^	1.04	5.43^***^	2.44^*^	−0.11
Traditional celebrities	1.68	1.87	3.64^***^	2.71^**^	1.04
Social media influencers	3.53^***^	0.95	3.89^***^	0.62	0.07
Food brands	3.77^***^	−1.07	5.33^***^	−0.21	−0.87

^*^p < 0.05, ^**^p < 0.01, ^***^p < 0.001.

#### 3.1.1 Non-core food social media messages by peers

Higher perceived volume of exposure to non-core food messages posted by peers was significantly associated with non-core food liking and with both descriptive and injunctive norms among adolescents, as indicated by Kendall’s rank-correlation tests (all *Z* > 2.4, all *ps* < 0.05), but not with perceived healthiness and eating intentions (see Table 2). Additionally, the multiple multivariate regression model showed that the perceived volume of exposure to non-core food messages by peers remain significantly associated with increased non-core food intake after including the control variables (*F*(5) = 5.9, Pillai = 0.035, *p* < 0.001, *η*^2^ = 0.05) (see Table 3; Supplementary material for the more details). These findings partially confirm the second hypothesis H2a as self-reported exposure to peer social media non-core food messages was positively associated with increased non-core food attitudes, perceived norms, and intake among adolescents.

**Table 3:** Kendall’s rank correlation coefficients depicting associations between perceived volume of exposure to core food messages (from different social media messengers) and core food related eating outcomes among adolescents.

Social media messenger	Food liking	Perceived healthiness	Descriptive norms	Injunctive norms	Intention to eat	Food literacy
Peers	3.98^***^	−2.67^**^	2.93^***^	−2.61^**^	1.91	3.55^***^
Traditional celebrities	3.28^***^	−2.42^*^	1.83	−0.99	0.78	4.59^***^
Social media influencers	3.67^***^	−2.43^*^	2.13^*^	−0.07	1.22	4.32^***^
Food brands	3.32^***^	−2.09^*^	1.49	−1.49	1.07	4.09^***^
Health organizations	2.11^***^	−0.37	−0.42	−0.03	1.50	3.62^***^

^*^p < 0.05, ^**^p < 0.01, ^***^p < 0.001.

#### 3.1.2 Non-core food social media messages by traditional celebrities

Exposure to non-core food messages posted by traditional celebrities only showed a significant association with descriptive and injunctive norms favoring non-core food among adolescents (all *Z* > 2.70, all *ps* < 0.01, see Table 2). Additionally, after controlling for covariates in a multiple multivariate regression model, higher exposure to traditional celebrities’ non-core food messages significantly predicted adolescents’ non-core food intake (*F*(5) = 3.7, Pillai = 0.022, *p =* 0.003, *η*^2^ = 0.03), see Supplementary Table 3. This partially supports hypothesis H2b as self-reported exposure to non-core food messages posted by traditional celebrities was positively associated with higher perceived norms and intake of non-core food among adolescents, but was not associated with attitudes and eating intentions.

#### 3.1.3 Non-core food social media messages by social media influencers

Adolescents’ who reported higher exposure to non-core food messages by social media influencers, also showed significantly higher liking and descriptive norms favoring non-core food (all *Z* > 3.50 all *ps* < 0.001). No significant associations were observed with perceived healthiness, injunctive norms or eating intentions, see Table 2. Furthermore, the multiple multivariate regression model showed that higher perceived volume of exposure to influencers’ non-core food messages on social media significantly predicted non-core food intake after including the control variables (*F*(5) = 3.7, Pillai = 0.022, *p* < 0.01, *η*^2^ = 0.03), see Supplementary Table 3. These findings partially confirm H2c and demonstrate that self-reported exposure to social media non-core food messages posted by social media influencers is positively associated with increased non-core food attitudes, norms and intake among adolescents.

#### 3.1.4 Non-core food social media messages by brands

Similar to messages posted by social media influencers, Kendall’s rank correlation tests showed that perceived volume of exposure to marketed social media food messages posted by food brands was only significantly associated with higher liking and descriptive norms of non-core food (all *Z* > 3.70, all *ps* < 0.001, see Table 2). However, the multiple multivariate regression model indicated that, after including the control variables, adolescents’ perceived volume of exposure to brands’ non-core food messages on social media did not significantly predict non-core food intake (*F*(5) = 1.0, Pillai = 0.007, *p =* 0.396, *η*^2^ = 0.01), see Supplementary Table 3. Accordingly, H2d, stating that higher self-reported exposure to non-core food messages by brands will be associated with increased non-core food attitudes, perceived norms, eating intentions and intake among adolescents, is only partially supported.

### 3.2 Core food social media sources and how they relate to food outcomes and perceptions

To answer the second part of RQ2, this section examines the association between eating outcomes and the perceived volume of exposure to core food messages posted by various social media messengers; namely peers, traditional celebrities, social media influencers, food brands and health organizations.

#### 3.2.1 Core food social media messages by peers

Kendall’s rank correlation tests showed that perceived volume of exposure to core food messages posted by peers was only positively associated with core food liking, descriptive norms as well as food literacy (all *Z* > 2.90, all *ps* < 0.001) among adolescents, see Table 3. At the same time, perceived exposure to these messages was significantly associated with lower perceived healthiness and weaker injunctive norms of core food (all *Z* < −2.6, all *ps* < 0.001) but was not associated with eating intentions. Furthermore, the multiple multivariate regression model demonstrated that, after including the control variables, reported exposure to peer’s core food messages was not significantly associated with increased reported core food intake (*F*(5) = 1.3, Pillai = 0.008, *p =* 0.268, *η*^2^ = 0.01), see Supplementary Table 4. These findings partially confirm H3a as self-reported exposure to social media core food messages posted by peers is only positively associated with increased core food liking and descriptive norms as well as higher food literacy among adolescents.

#### 3.2.2 Core food social media messages by traditional celebrities

Kendall’s rank correlation tests showed that increased self-reported exposure to celebrities’ social media core food messages was significantly associated with greater liking of core food as well as higher food literacy scores (all *Z* > 3.20, all *ps <* 0.001), yet with lower perceived healthiness of core food (*Z* = −2.42, *p* < 0.05), see Table 3. No significant associations were observed with perceived norms or eating intentions. Additionally, the multiple multivariate regression model demonstrated that, after accounting for the control variables, perceived volume of exposure to traditional celebrities’ core food messages was not significantly associated with increased core food intake among adolescents (*F*(5)= 0.6, Pillai = 0.004, *p* = 0.685, *η*^2^ = 0.00), see Supplementary Table 4. These findings partially support H3b as self-reported exposure to social media core food messages posted by traditional celebrities was positively associated with increased core food liking and food literacy among adolescents.

#### 3.2.3 Core food social media messages by social media influencers

Increased self-reported exposure to social media influencer’s core food messages on social media was significantly associated with higher core food liking and descriptive norms as well as greater food literacy, as indicated by Kendall’s rank correlation tests (all *Z* > 2.10, all *ps* < 0.05), yet associated with lower perceived healthiness of core food (*Z* = −2.43, *p* < 0.05), see Table 3. No significant associations was observed with injunctive norms or eating intentions. Similar to reported exposure to social media core food messages posted by peers, and traditional celebrities, self-reported exposure to influencers’ core food messages on social media does not seem to significantly predict core food eating (*F*(5) = 1.1, Pillai = 0.007, *p =* 0.368, *η*^2^ = 0.00) in the adjusted multiple multivariate regression model, see Supplementary Table 4. These findings partially support H3c indicating that perceived exposure to core-food messages posted by influencers is positively associated with adolescents’ core-food liking and norms as well as food literacy, but does not translate into higher core-food intake.

#### 3.2.4 Core food social media messages by brands

Similar to patterns observed for peers, celebrities and influencers, adolescents who reported higher exposure to food brand’s core food messages had significantly higher core food liking and greater food literacy, as indicated by Kendall’s rank correlation tests (all *Z* > 3.30, all *ps* < 0.0001), yet lower perceived healthiness of core food (*Z* = −2.09, *p* < 0.05), see Table 3. Additionally, perceived volume of exposure to core food messages by brands did not significantly predict core food eating (*F*(5) = 1.4, Pillai = 0.009, *p =* 0.239, *η*^2^ = 0.01) in the adjusted multiple multivariate regression model, see Supplementary Table 4. These findings partially support H3d as self-reported exposure to social media core food messages posted by food brands was positively associated with increased core food liking and food literacy.

#### 3.2.5 Core food social media messages by health organizations

Although not part of our hypothesis, we also explored the association between self-reported exposure to core food messages by health organizations and eating outcomes among adolescents. As presented in Table 3, perceived volume of exposure to core food messages posted by health organizations was significantly associated with increased liking of core food and higher food literacy (all *Z* > 2.10, all *ps* < 0.0001). Unlike reported exposure to core food messages posted by peers, traditional celebrities, influencers, and brands, self-reported exposure to health organizations’ core food messages on social media remained significantly associated with increased core food eating (*F*(5) = 3.0, Pillai = 0.023, *p =* 0.012, *η*^2^ = 0.02) when including control variables in a multiple multivariate regression model, see Supplementary Table 4 for more details.

### 3.3 Health organizations and peers have the strongest associations with core and non-core food intake among adolescents

The third research question of this study asked which social media messengers, in terms of adolescents’ perceived volumes of exposure to core and non-core food messages, had the strongest association with core and non-core food intake among adolescents. To investigate RQ3 and compare the different social media messengers, we built two multiple multivariate regression models. The first model (see Table 4) demonstrated that perceived volume of exposure to non-core food messages posted by peers is the strongest predictor of non-core food intake, when controlling for gender, self-regulation, BMI-for-age, and intentions to eat non-core food (*F*(5) = 5.7, Pillai = 0.037, *p <* 0.001, *η*^2^ = 0.03). This also qualifies the findings for RQ1. The second model (Table 5) demonstrated that perceived volume of exposure to core food messages posted by health organizations is the strongest and only significant predictor of core food intake, after controlling for other covariates (*F*(5) = 2.9, Pillai = 0.022, *p =* 0.013, *η*^2^ = 0.02).

**Table 4:** The Pillai values and eta squares of perceived volumes of exposure to different sources of non-core food messages on social media in a multiple multivariate regression model predicting self-reported non-core food intake measures among adolescents.

Variable	Pillai	*η* ^2^
Peers	0.037^***^	0.03
Traditional celebrities	0.008	0.01
Social media influencers	0.010	0.00
Food brands	0.005	0.01

^**^p < 0.01, ^***^p < 0.001.

**Table 5:** The Pillai values and eta squares of perceived volumes of exposure to different sources of core food messages on social media in a multiple multivariate regression model predicting self-reported core food intake measures among adolescents.

Variable	Pillai	*η* ^2^
Peers	0.015	0.02
Traditional celebrities	0.005	0.01
Social media Influencers	0.013	0.01
Health organizations	0.022^*^	0.02

^*^p < 0.05.

## 4 Discussion

Previous research demonstrates that increased self-reported exposure to food (marketed) messages on social media is associated with increased reported food intake as well as eating attitudes, perceived norms and food literacy among adolescents (34). However, this research did not investigate the power of the message or differentiate between the different sources of these social media food messages. Accordingly, this study addresses this gap and demonstrates how exposures to social media food messages communicated by different sources (i.e., peers, traditional celebrities, social media influencers, brands, and heath organizations), differ and compare in their association with eating outcomes among adolescents between 11–19 years of age. Several findings regarding non-core food message sources are highlighted here: First, increased self-reported exposure to non-core food messages, regardless of the source, was consistently associated with higher perceived descriptive norms favoring non-core food, where adolescents reported their perceptions that their peers consume much non-core food. Second, non-core food messages posted on social media by peers, traditional celebrities, influencers, and brands were largely associated with increased liking for and/or consumption of non-core foods. Third, self-reported volume of exposure to non-core food messages posted by peers showed the strongest association with non-core food intake, as compared to perceived exposure to (marketed) messages by, brands influencers, and celebrities.

Regarding core food message exposure and adolescents’ eating outcomes, the following findings are highlighted: First, overall exposure to core food messages, regardless of the source, was consistently associated with higher liking of core food, as well as food literacy. Second, peers and health organizations as sources of core food messages stood out in terms of their associations with positive core food outcomes. Adolescents’ perceived volumes of exposure to core food messages posted by peers were positively linked with increased perceived norms favoring core food, while perceived volume of exposure to messages by health organizations significantly predicted core food intake among adolescents in the multiple multivariate regression models. Across all messenger types, perceived exposure to both non-core and core food messages was not associated with adolescents’ intentions to eat these foods.

Findings of this study confirm the importance of peers in shaping adolescents’ eating behavior, especially non-core food eating outcomes. This is in accordance with previous studies that have highlighted the significance of peers in influencing food intake among adolescents in real-life physical settings, particularly concerning the encouragement of non-core food consumption (15, 52). The present study shows that the same is true in the context of social media, where adolescents spend great amounts of time interacting with peers, which is the DNA of social media. The opportunities for peer influence on social media are high. Social media properties, such as number of likes, serve as social endorsements by peers and facilitate peer’s influence on adolescent’s relationship with food (52). Furthermore, adolescents’ reported exposure to great volumes of non-core food messages posted by their peers was in stark difference to that of core food message exposure by peers. In fact, this difference was larger than any other difference found between reported exposure to core and non-core food messages by traditional celebrities, social media influencers and brands. This finding aligns with studies that found adolescents often share non-core food messages on social media, of which much is marketed (11, 12). This high exposure to non-core food messages, in line with the mere exposure phenomenon, further enhances the persuasiveness of the communicated—including marketed—non-core food message, increasing adolescents’ attitude towards and intake of non-core food. As such, adolescents seem to be acting as influencing agents among each other, greatly persuading one another to prefer and consume non-core food.

Self-reported exposures to non-core food messages by traditional celebrities and social media influencers seem to be similar in their association with the different assessed eating outcomes, particularly in their effect size on non-core food intake. This finding agrees with literature that identified social media influencers and traditional celebrities as significant factors in influencing children’s eating outcomes. For example, Boyland et al. (53) found that pre-adolescents increased their consumption of non-core food following exposure to celebrity endorsed television commercials. Similarly, Coates et al. (54) found that exposure to non-core food messages by (mock) social media influencers increased the non-core food consumption of pre-adolescents. In the current study, we see that non-core food messages by celebrities and influencers follow similar paths and are equal in terms of their association with adolescents eating. We expect that evaluative conditioning plays a role in these associations where the pairing of the non-core food with the celebrity or influencer leads to a transfer of the valence of the messenger to the food communicated in the message (54).

However, similarities between celebrities and influencers seem to fade when the food addressed is core. In this context, social media influencers seemed to have stronger associations with core food outcomes than celebrities. Several explanations can be adopted. It is common for celebrities and influencers, especially those whose accounts are not devoted to food, to post about food (with non-core food being the dominant message). However, certain influencers post solely about core food, as their accounts are devoted to these foods or a healthy lifestyle (22). This is less common among traditional celebrities. Furthermore, social media influencers who post about core food are typically perceived as experts in core food as a health subject, a lifestyle, or a cooking approach (19). As McGuire (9) predicted in his Communication/Persuasion model, this perceived messenger expertise is likely to improve the persuasiveness of food messages. Yet at the same time, increased perceived exposure to these influencers’ core food messages, similar to patterns observed for celebrities, peers and brands, was associated with decreased perceived healthiness of core food. This could be related to the positive perceptions of these messengers creating negative evaluations of the food they promote (i.e., a reversed halo effect).

Although social media food messages by peers, celebrities, and influencers typically include marketed messages, these food messages are often covertly marketed requiring persuasion knowledge to identify their marketing intent (55). In comparison, marketed food messages posted by brands are typically more overtly marketed and are probably more easily identified by adolescents as marketed messages. The Persuasion Knowledge Model predicts that an adolescent who identifies the marketing intent of a message will be able to employ the coping skills necessary to resist this message (55). As such, this could mean that those who identified exposure to food content posted by brands were mostly reporting on overt marketing content and were also more likely to resist the persuasiveness of these messages. Indeed, the present study shows that those who reported higher exposure to non-core food messages posted by brands also reported higher liking of core food, and higher intake of some core foods. However, this exposure did not significantly predict adolescent’s non-core food intake in the multivariate models, which could indicate that adolescents are employing some type of resistance to the message. Another explanation for the lack of association between exposure to branded food and intake among adolescents, could align with the *two-factor theory by* Berlyne (56). According to this theory, repeated exposure to a food message initially results in a favorable response to the message up until a point where any further repetition of the message creates boredom and inattention to the message. Accordingly, an increased repetition in a marketed food message could create a U-shaped course of effects, where an adolescent initially has positive attitudes to the message, but with increased repetition these attitudes weaken or the persuasive impact diminishes (56, 57). This may help explain why exposure to branded food messages was associated with some positive attitudinal outcomes but did not translate into higher non-core food intake.

The finding that core food messages by health organizations are significantly associated with increased eating outcomes favoring core food, including food literacy, liking, and intake, is promising. There are several possible explanations for this finding. First, it is possible that the messages shared by health organizations are more accurate, stronger in language, and more focused on core food. Second, it is possible that health organizations are perceived as a more credible source of nutritional information as compared to other sources such as peers and traditional celebrities (26). In contrast, peers are not typically viewed as credible health sources, which could explain why the weaker injunctive norms observed for peer posts. According to the Communication/Persuasion model, this combination of a more focused and stronger message (as a message characteristic) that is communicated by a credible messenger (as a source characteristic) facilitates the persuasiveness of the message communicated to adolescents (9, 10). Another possible explanation for the link between health organizations and core food consumption, is that those who are more interested in healthy core food eating are simply more likely to deliberately seek social media posts by health sources such as health organizations.

This secondary analysis of a previously published dataset offer complementary evidence to persuasion research on audience characteristics. Similar to message and source factors, persuasion research demonstrates that audience characteristics shape how strongly individuals respond to persuasive content. Adolescents are not a homogeneous audience; developmental stage, gender, and individual traits shape how food messages and marketing are processed and acted upon. For example, Murphy et al. (22) found that adolescents’ attention to unhealthy food advertising increases with age, indicating that older adolescents may be potentially more susceptible to non-core food marketed messages despite being more cognitively developed. Gender differences in response to food marketed messages have also been observed (58) while some personality traits can heighten adolescents’ susceptibility to persuasive content (30). Although our dataset did not include detailed psychological measures, we accounted for demographic and individual characteristics such as gender and self-regulated autonomy. In this study, even after adjusting for these audience factors, adolescents’ perceived exposure to different social media messengers remains strongly associated with food intake. These insights also highlight the need for future research to examine how specific audience traits interact with message- and source-related processes across the diverse developmental span of adolescence.

This study is not without limitations. This study focused on self-reported measures, which introduce response bias. Another bias is the difficulty and error possibly associated with asking participants to differentiate between peers, social media influencers, traditional celebrities, brands, and health organizations. To limit such errors and difficulty, we provided participants with standard written explanations of our working definitions of peers, influencers, and traditional celebrities. We also conducted cognitive interviews to test the face-validity of these terms and ensure that adolescents understand the questions as intended by the researchers. Furthermore, we used broad intake categories (aligned with validated food frequencies, see Matthys et al. (41)) to reduce respondent burden in a school-based survey; however, this may also reduce sensitivity to food or product-specific preferences. Perceived exposure was assessed without an explicit timeframe, which may introduce variability in how participants construed the reference period. Nevertheless, within-person, relative comparisons remain interpretable because respondents rely on a shared internal calibration across item types. Furthermore, a fixed window (e.g., past week/month) would have been challenging given the heterogeneity of exposures to different foods, some of which are encountered rarely and others frequently. Finally, we do not know whether the exposure assessed in this study is incidental or consciously sought for. Despite the limitations of this research, this is one of the first studies to our knowledge to compare the social media food messages between different messengers—namely peers, celebrities, influencers, brands and health organizations—in association with adolescent eating. While the data was collected in 2019, since then there have been increasing research on social media senders, most of it focusing on influencers (e.g., see 3, 19, 54, 59), further highlighting the relevance of this secondary analysis.

The findings of this study hold various practical implications. Messages from peers showed strongest associations with self-reported food intake. One must not forget that the majority of food messages posted by peers on social media, including marketed messages, promote non-core foods (11). Hence, this leaves the incorporation and empowerment of peers in health promotional efforts as a possible avenue to explore. Furthermore, the positive association between self-reported exposure to peer’s core food messages on social media and higher liking of core food and less liking of non-core food is promising for health interventions and campaigns. These findings suggest that incorporating social media peers in health promotion may be an important element to break the non-core food norm sharing pattern on social media and increase the popularity and consumption of core food in real-life. This also stresses the importance of implementing and reinforcing policies targeting non-core food brands that currently incorporate adolescent peers in their marketing strategies, for instance by encouraging adolescents to engage with fan pages, launching competitions promoting non-core food brands and products, and encouraging reposts of branded non-core food content. There is a need for policies that are not self-regulatory and that are timely and well suited to protect adolescents from current and new emerging social media food marketing strategies. These regulations should also cover celebrity and influencer endorsements of non-core food on social media. Health organizations and public health campaigns should make use of social media as a free and effective platform for health communication. We also call for future research to include experiments that study the causal effect of social media messengers, including the ones investigated in this study, on eating outcomes among adolescents. The associations found in this cross-sectional study could also be in part related to the differences in the characteristics of messages, including how the messages are shaped and what content they include. Hence, we call for future experiments to investigate whether a message, identical in its content and all other message characteristics, but presented by different sources would have a similar or different impact on adolescent eating outcomes. A comprehensive body of research regarding food messages and messengers is essential in guiding health professional efforts in the promotion of core food among adolescents.

## Data Availability

The datasets presented in this study can be found in online repositories. The names of the repository/repositories and accession number(s) can be found at: https://osf.io/cxk8q/?view_only=c1d36fd4b603456da84ee9cfd61aac33.
