# Supplementary material for: Who sends the message matters: social media messengers and adolescent eating
Source: Front Nutr. 2026 May 4;13:1799978. doi: 10.3389/fnut.2026.1799978 (PMC13180906; doi:10.3389/fnut.2026.1799978)
Supplement: Supplementary file 1 [file Supplementary_File_1.docx]

Supplementary Material

**Additional results on the relationship between perceived volume of exposure to food messages and adolescent eating:**

***Exposure to non-core food***

Perceived volume of exposure to non-core food messages posted by peers showed the strongest association with intake of savory non-core snacks such as chips (Z = 2.79, p = 0.005) and the second strongest association with intake of sweet non-core snacks such as soft drinks (Z = 2.67, p = 0.007). Kruskal Wallis tests also showed that increased self-reported exposure to peer’s non-core food messages on social media was significantly associated with increased junk food intake (*H*(5) = 32.64, *p* < .001). Furthermore, Kruskal-Wallis tests and Kendall’s rank correlations revealed that increased self-reported exposure to celebrities’ non-core food messages was significantly associated with higher self-reported non-core food intake among adolescents, including sugar (H(6) = 15.79, p = 0.015), savory non-core snacks (Z = 2.12, *p =* 0.034), and junk food intake (H(5) = 16.94, *p =* 0.005). However, these non-core messages were also associated with increased self-reported intake of fruits (Z = 1.96, p = 0.05). Kendall’s rank correlation and Kruskal Wallis tests showed that increased self-reported exposure to social media influencers’ non-core food messages was significantly associated with higher intake of non-core food, including sweet snacks such as candy and chocolate (Z = 2.40, p = 0.016), savory snacks (Z = 2.46, *p =* 0.014), and junk food (H(5) = 19.84, *p =* 0.001), higher food literacy (Z=2.27, p = 0.023) and intentions to eat core food (Z= 3.43, p < .001). As for brands, adolescents who reported increased exposure to non-core food messages posted by brands, were significantly more likely to report non-core food intake including sweet snacks (Z = 2.725, *p =* 0.006), savory snacks (Z = 1.917, *p =* 0.055), as well as core food intake including fruits (Z = 2.210, *p* = 0.027), and vegetables (Z = 3.024, *p =* 0.002).

***Exposure to core food***

Kendall’s rank correlation tests showed that increased self-reported exposure to celebrities’ core food messages on social media was significantly associated with higher intake of core food, specifically vegetables (Z = 2.47, *p =* 0.014). Interestingly, Kendall’s rank correlation tests also showed that increased self-reported exposure to celebrities’ social media core food messages was significantly associated lower perceived healthiness of core food (Z = -2.42, p = 0.016) and with higher perceived healthiness (Z = 2.36, p = 0.018) of non-core food.

Kendall’s rank correlation tests showed that increased self-reported exposure to social media influencer’s core food messages on social media was significantly associated with higher liking of core food (Z=3.67, p < .001) and lower liking of non-core foods (Z= -3.24, p = 0.001). Furthermore, increased perceived volume of exposure to influencers’ core food messages on social media was significantly associated with higher intake of vegetables (*Z* = 2.41, p = 0.016) and fruits (*Z* = 2.12, p = 0.034), and lower intake of non-core food including sweet spreads such as chocolate spread and syrup (Z = -2.12, p = 0.034) and sugared drinks such as soft drinks (*Z* = -2.28, p = 0.023).

Based on Kendall’s rank correlation tests, adolescents who reported higher exposure to brand’s core food messages had significantly lower perceived healthiness of core food (Z = -2.09, p = 0.036) and higher perceived healthiness of non-core food (Z = 1.98, p = 0.048). Additionally, Kendall’s rank correlation tests showed that increased self-reported exposure to food brand’s core food messages on social media was significantly associated with higher intake of core food, specifically vegetables (Z = 2.64, p = 0.008), fruits (Z = 2.09, p = 0.037), and beans (Z = 3.66, *p* < .001). A Kruskal-Wallis tests also showed that increased reported exposure to branded core food messages was significantly associated with increased junk food intake (H(5) = 11.52, p = 0.042). As for health organizations, a Kendall’s rank correlation tests showed that increased perceived volume of exposure to health organizations’ core food messages on social media was significantly associated with higher intake of core foods: fruits (Z = 3.05, p = 0.002) and beans (Z = 4.29,*p* < .001).
