# Supplementary material for: Who sends the message matters: social media messengers and adolescent eating
Source: Front Nutr. 2026 May 4;13:1799978. doi: 10.3389/fnut.2026.1799978 (PMC13180906; doi:10.3389/fnut.2026.1799978)
Supplement: Supplementary file 2 [file Table_1.docx]

Supplementary Material

Table 1 Differences in adolescents’ reported exposure to core food messages across message source types based on sign test analyses (S and p values)

| Comparison | Difference in exposure between sources |
| --- | --- |
| Traditional celebrities vs. Peers | S = 595, p < .001 |
| Traditional celebrities vs. Social Media Influencers | S = 351, p = .066 |
| Traditional celebrities vs. Food brands | S = 308, p < .001 |
| Traditional celebrities vs. Health Organizations | S = 210, p < .001 |
| Social Media Influencers vs. Peers | S = 585, p < .001 |
| Social Media Influencers vs. Health Organizations | S = 195, p < .001 |
| Food brands vs. Peers | S = 636, p < .001 |
| Food brands vs. Health Organizations | S = 236, p < .001 |
| Health Organizations vs. Peers | S = 559, p < .001 |
