# Supplementary material for: Who sends the message matters: social media messengers and adolescent eating
Source: Front Nutr. 2026 May 4;13:1799978. doi: 10.3389/fnut.2026.1799978 (PMC13180906; doi:10.3389/fnut.2026.1799978)
Supplement: Supplementary file 3 [file Table_2.docx]

Supplementary Material

Table 2 Differences in adolescents’ reported exposure to non-core food messages across message source types based on sign test analyses (S and p values)

| Comparison | Difference in exposure between sources |
| --- | --- |
| Traditional celebrities vs. Peers | S = 421, p = .518 |
| Traditional celebrities vs. Social Media Influencers | S = 314, p < .001 |
| Traditional celebrities vs. Food brands | S = 202, p < .001 |
| Social Media Influencers vs. Peers | S = 440, p = .137 |
| Food brands vs. Peers | S = 565, p < .001 |
| Food brands vs. Social Media Influencers | S = 578, p < .001 |
