# Supplementary material for: Who sends the message matters: social media messengers and adolescent eating
Source: Front Nutr. 2026 May 4;13:1799978. doi: 10.3389/fnut.2026.1799978 (PMC13180906; doi:10.3389/fnut.2026.1799978)
Supplement: Supplementary file 4 [file Table_3.docx]

Supplementary Material

Table 3 Pillai values of food intake predictors in the non-core food exposure (by peers, traditional celebrities, social media influencers, and brands) multiple multivariate regression models

| Variable  Model | Food Messages | Gender | TSR | Intention to eat | BMI-for-Age |
| --- | --- | --- | --- | --- | --- |
| Peers | 0.035*** | 0.114*** | 0.047*** | 0.006 | 0.011 |
| Traditional celebrity | 0.022** | 0.099*** | 0.046*** | 0.005 | 0.011 |
| Social media Influencers | 0.022** | 0.100*** | 0.047*** | 0.005 | 0.012 |
| Brands | 0.007 | 0.083*** | 0.045*** | 0.004 | 0.009 |

** *p*<.01, ****p*<.001
