# Supplementary material for: Who sends the message matters: social media messengers and adolescent eating
Source: Front Nutr. 2026 May 4;13:1799978. doi: 10.3389/fnut.2026.1799978 (PMC13180906; doi:10.3389/fnut.2026.1799978)
Supplement: Supplementary file 5 [file Table_4.docx]

Supplementary Material

Table 4 The Pillai values of the food intake predictors in the core food exposure (by peers, traditional celebrities, social media influencers, and brands) multiple multivariate regression models

| Variable  Model | Food Messages | Gender | TSR | Intention to eat | BMI-for-Age |
| --- | --- | --- | --- | --- | --- |
| Peers | 0.008 | 0.054*** | 0.019** | 0.010 | 0.032*** |
| Traditional celebrity | 0.004 | 0.050*** | 0.019** | 0.009 | 0.032*** |
| Social media Influencers | 0.007 | 0.048*** | 0.017* | 0.012 | 0.032*** |
| Brands | 0.009 | 0.055*** | 0.019* | 0.010 | 0.027*** |
| Health organizations | 0.023* | 0.052*** | 0.028** | 0.020* | 0.024** |

**p* < .05, ***p* < .01, ****p* < .001
